# Periodontitis severity and its social and clinical determinants: An ACES framework‐based NHANES analysis

**DOI:** 10.1002/jper.70064

**Published:** 2026-01-19

**Authors:** Meng Xuan Chen, Yue Yu, Chen Xuan Wei, Hamoun Sabri, Muhammad H. A. Saleh

**Affiliations:** ^1^ Department of Periodontics and Oral Medicine University of Michigan School of Dentistry Ann Arbor Michigan USA; ^2^ Department of Mechanical & Industrial Engineering University of Toronto Toronto ON Canada; ^3^ Department of Biologic and Materials Sciences and Prosthodontics University of Michigan School of Dentistry Ann Arbor Michigan USA

**Keywords:** classification, periodontitis, poverty, prevalence, socioeconomic factors

## Abstract

**Objectives:**

To assess associations between the poverty‐income ratio (PIR) and periodontitis severity using Application of the 2018 Periodontal Status Classification to Epidemiological Survey Data (ACES) framework.

**Methods:**

Three NHANES cycles (2009–2014) with adults aged ≥30 years with complete periodontal examinations (*n* = 10,598) were included. Staging (I–IV), grading (A–C), and extent (localized/generalized) were derived. The primary exposure was the poverty‐income ratio. The covariates included smoking (current/former/never), HbA1c, and BMI. The analytic sample for the PIR main effects was *n* = 9708. Stage/grade used multinomial logistic regression; extent used binary logistic regression. Full models included the main effects and interactions; non‐significant interactions were removed. Significance was set at *p *< 0.05. Analyses were performed in R.

**Results:**

A higher PIR was protective across outcomes: stage IV versus I RRR = 0.58, stage III 0.73; and stage II 0.87 (*p *< 0.001); grade C versus A 0.74, grade B 0.83 (*p *< 0.001); generalized extent OR = 0.93 (*p *< 0.001). Smoking showed a graded risk: current versus never – stage III RRR = 2.19, stage IV 3.82 (*p *< 0.001); grade B 1.57, grade C 2.18 (*p *< 0.001); generalized OR = 1.46 (*p *< 0.001); former versus never generalized OR not significant (*p *= 0.24). The HbA1c increases were associated with a higher severity: stages II–IV versus I RRRs = 1.56/1.97/2.14 (all *p *< 0.001); grades B/C versus A 1.58/1.82 (*p *< 0.001); extent OR = 1.00 (*p *= 0.81). With BMI models, the BMI showed slight associations for stage/grade (RRR≈0.93–0.98), while the PIR remained strongly associated: stage II/III/IV RRRs = 0.69/0.48/0.30 (all *p *< 0.001); grade B/C 0.54/0.43 (*p *< 0.001); extent OR for PIR = 0.93 (*p *< 0.001), BMI OR = 1.00 (*p *= 0.184).

**Conclusions:**

Within the limitations of this cross‐sectional analysis, a higher PIR was associated with a lower periodontitis severity, smoking, and higher HbA1c with higher severity, and the BMI effects were minimal.

**Plain language summary:**

Gum disease is common and tied to overall health. People with less income and consequently fewer resources for preventative care may face a higher risk for disease. We analyzed three cycles of the US National Health and Nutrition Examination Survey (2009–2014). Adults aged ≥30 years with complete dental examinations (*n* = 10,598) were classified using the 2018 staging/grading system that defines disease severity (stage) and the risk for progression (grade). Socioeconomic status was measured by the poverty‐income ratio (PIR). We examined links between PIR and disease stage, grade, and whether the disease was localized or generalized, and considered smoking, blood sugar (HbA1c), and body mass index (BMI). Our results demonstrated that a higher PIR (more resources) was consistently linked to less severe and less widespread disease. Current smoking showed strong, stepwise increases in severity and spread; former smoking was not linked to spread. A higher HbA1c was tied to more severe stages and grades, but not to spread. The BMI showed only small, inconsistent effects. Overall, lower income, smoking, and poor blood‐sugar control tracked with worse periodontal status.

## 1 INTRODUCTION

Periodontitis is a global public health challenge, affecting over 1 billion cases worldwide.^1^ In the United States (US), periodontitis affects an estimated 42% of dentate adults aged 30 years and older.^2^ Periodontitis, classified as a chronic non‐communicable disease (NCD), is linked to systemic health conditions, and shares common risk factors with other NCDs.^3^ As a NCD, periodontitis can be managed through risk factor modification.^3^

Given these established relationships, the National Health and Nutrition Examination Survey (NHANES) serves as the principal resource for assessing periodontal disease patterns of the US population. Previous studies have documented periodontitis as a highly prevalent oral disease among US adults and have established descriptive patterns by demographic characteristics including age, sex, and race/ethnicity.^2^, ^4^ However, comprehensive risk factor analysis, particularly regarding socioeconomic determinants, remains limited. In 2018, a classification framework for periodontitis was developed to better evaluate disease severity, complexity, extent, and progression.^5^, ^6^

To ensure consistency and comparability in epidemiological research, this study applied the “Application of the 2018 Periodontal Status Classification to Epidemiological Survey data” (ACES) framework proposed by Holtfreter et al.^7^ This framework specifies standardized criteria for staging, grading, and extent assessment in population‐based surveys, enabling alignment with recent epidemiological applications of the 2018 classification.^5^, ^6^

While recent research has applied the ACES periodontal classification system to NHANES data,^8^ comprehensive risk factor analyses remain limited. Among modifiable risk factors, socioeconomic status has emerged as a significant risk indicator for periodontitis.^9^, ^10^, ^11^, ^12^ Globally, higher socio‐demographic indices are associated with lower age‐standardized prevalence rates across countries.^9^ In the US, previous studies have consistently demonstrated an inverse relationship between periodontitis and socioeconomic positions.^10^, ^11^, ^12^ This association might be explained through established periodontal risk factors, as a low socioeconomic status is significantly associated with a higher prevalence of cigarette smoking,^13^ an increased likelihood of diabetes diagnosis,^14^ and a greater risk of obesity.^15^

Therefore, this study aims to investigate the association between socioeconomic status and periodontitis using the new classification framework, and to evaluate the interplay between established factors – smoking, diabetes, obesity – and socioeconomic status in periodontitis status.

## 2 METHODS

### 2.1 Study population

This cross‐sectional study followed the STROBE guidelines.^16^ All data were extracted from the National Health and Nutrition Examination Survey (NHANES) database utilizing three combined cycles (2009–2010; 2011–2012; and 2013–2014) to maximize the sample size and statistical power. All data were obtained from the US Centers for Disease Control and Prevention (CDC) website (http://www.cdc.gov/nchs/nhanes/). NHANES uses a complex, multistage, stratified probability sample of the civilian, noninstitutionalized US population with oversampling of key subgroups. These cycles included full‐mouth periodontal examinations for adults aged ≥30 years with measurements recorded at six sites per tooth. Examinations were conducted in mobile examination centers (MECs) using automated, computerized data entry. Quality assurance included examiner training, calibration, periodic monitoring, and annual retraining by a reference examiner. Data were systematically checked for inconsistencies and errors to ensure high quality. Oral health data were released in separate component files, and merging across three cycles was performed to increase the analytic power. This present study applied the 2018 Periodontal Status framework to participants aged ≥30 years with complete periodontal examinations, yielding 10,598 patients with full stage (I–IV), grade (A–C), and extent (generalized/localized) classifications. This study is a secondary analysis of publicly available, de‐identified NHANES datasets and therefore did not require institutional ethics committee/IRB review. NHANES data collection protocols were reviewed and approved by the NCHS Ethics Review Board, and participants provided informed consent.

### 2.2 Data collection

We restricted the sample to participants with a complete full‐mouth periodontal examination and assignable ACES stage/grade/extent (*n* = 10,598). For primary analyses, we required a non‐missing poverty‐income ratio (PIR) and excluded records with missing PIR (analytic *n* = 9708). For models including additional covariates, we used complete‐case analysis per model (no statistical imputation): smoking status (*n* = 9699), diabetes status assessed through HbA1c values (*n* = 9376), and obesity determined by BMI values (*n* = 9854). Smoking was classified as current (≥100 lifetime cigarettes + current use), former (≥100 lifetime cigarettes + no current use), or never smokers.^17^ HbA1c was measured by high performance liquid chromatography from blood specimens collected during the MEC visit and analyzed as a continuous variable. BMI was calculated as a continuous variable from the measured height and weight (BMI = weight in kg/height in m^2^) obtained during the physical examination. To reduce any missingness in grade modifiers, self‐reported smoking was supplemented by plasma cotinine where available; diabetes status was supplemented by HbA1c. If both self‐report and the corresponding biomarker were unavailable, the grade was based on indirect CAL/root‐length estimates without modifier up‐rating. Participants not meeting the diagnostic criteria were classified as non‐periodontitis.

The demographic measures collected included smoking status (never, former, current) and the family income‐to‐poverty ratio (continuous from 0 to 5). Clinical measures encompassed HbA1c (%) and BMI (kg/m^2^). Periodontal measures included stage (I–IV), grade (A–C), and extent (localized/generalized), classified using the 2018 ACES criteria.^7^

Periodontitis was identified when the participants had either interdental clinical attachment loss (CAL) of ≥1 mm at two or more non‐adjacent sites, or buccal/oral CAL of ≥3 mm with a probing pocket depth (PPD) of ≥3 mm at two or more teeth. Staging followed these criteria: stage I, maximum interdental CAL 1–2 mm; stage II, maximum interdental CAL 3–4 mm without PPD ≥6 mm at two or more non‐adjacent sites; stage III, maximum interdental CAL 3–4 mm with PPD ≥6 mm at two or more non‐adjacent sites, or CAL ≥5 mm with ≥20 remaining natural teeth; stage IV, CAL ≥5 mm with <20 remaining natural teeth. The PPDs at the distal surfaces of second molars were excluded. Furcation involvement and vertical bone loss were not applied due to unavailable data.

Grading was determined using indirect progression measures (relative CAL as 100 × CAL (mm)/root length (mm) per the ACES framework),^7^ followed by adjustments according to grade modifiers by self‐reported daily smoking and HbA1c levels in individuals reporting diabetes.^5^ The root length was assigned based on the sex and ethnicity of each subject using data from previous publications.^18^, ^19^, ^20^, ^21^, ^22^, ^23^ Since data regarding each ethnicity in the US is unavailable, we combined ethnicities into four more categories (White, Black, Asian, and Hispanic). Missing smoking or diabetes data were supplemented with plasma cotinine and HbA1c values, respectively. If neither self‐reported nor biochemical measures were available, the original grade based on CAL estimations was retained. Participants not meeting the diagnostic criteria were classified as non‐periodontitis cases.

### 2.3 Statistical analysis

The analysis proceeded in two stages using the PIR as the primary socioeconomic indicator. The PIR represents a more reliable measure of socioeconomic status than education or occupation, accounting for household size, cost of living, and consumer price index relative to income and federal poverty thresholds.^24^ In NHANES, PIR is a predetermined continuous variable ranging from 0 to 5, with lower values indicating lower socioeconomic status.^25^ Initially, associations between the PIR and periodontal outcomes (stage, grade, extent) were examined in 9708 participants. Subsequently, the effects of PIR in combination with covariates – smoking status (categorical), HbA1c (continuous), and BMI (continuous) – were assessed to explore the potential effect modification. Sample sizes varied by covariate availability (smoking: *n* = 9699; diabetes: *n* = 9376; obesity: *n* = 9854). Ordinal logistic regression for stage and grade outcomes violated proportional odds assumptions (Brant tests), requiring multinomial logistic regression. Binary logistic regression was used for the dichotomous extent outcome. Full models containing all the main effects and interactions were fitted, with non‐significant interaction terms subsequently removed. All analyses were performed using R software. Statistical significance was assessed at *p* < 0.05.

## 3 RESULTS

**FIGURE 1:**
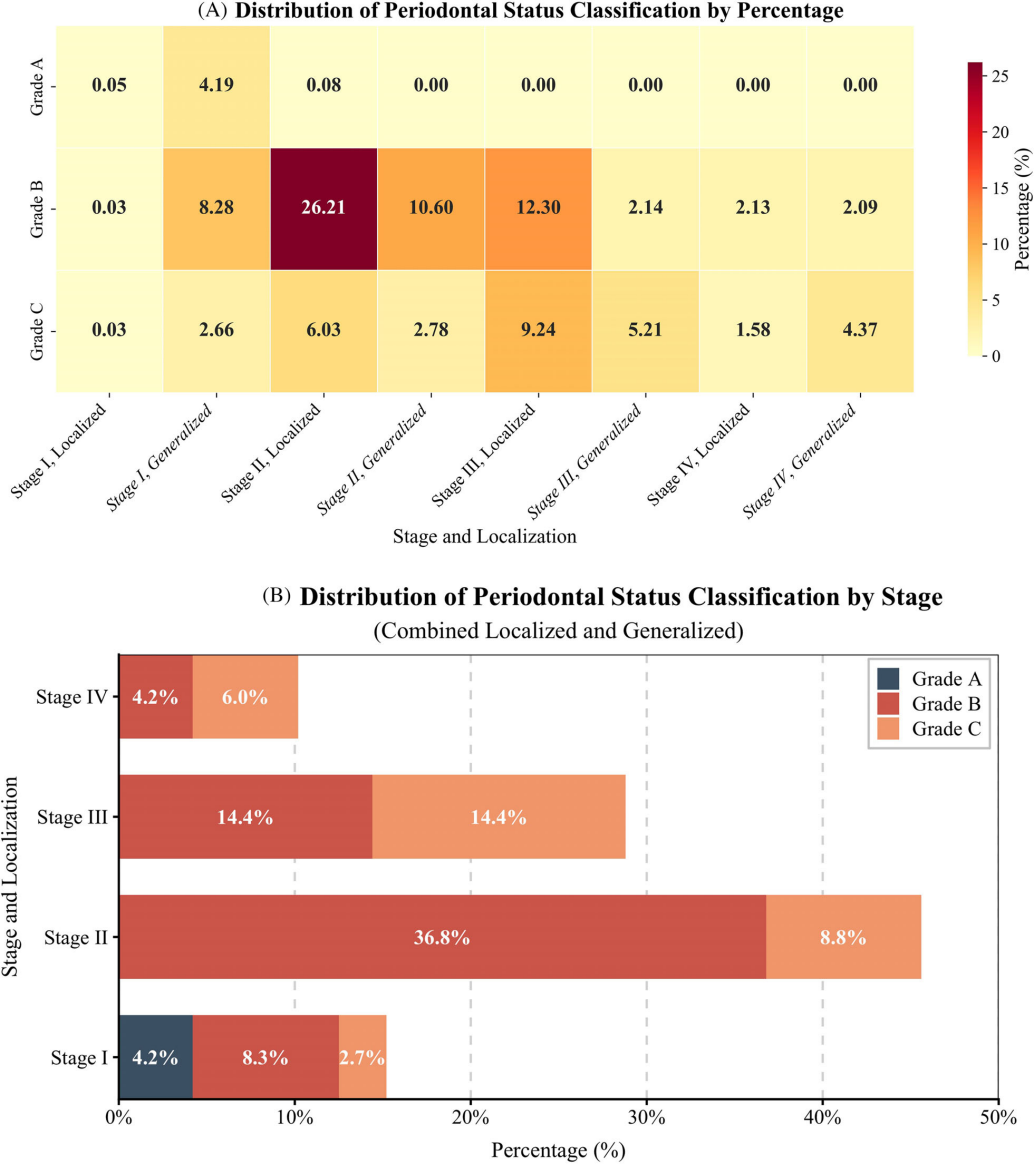
(A) Distribution of periodontal status classifications by percentage according to stage (I–IV), grade (A‐C), and extent (localized/generalized) in the study population. Each cell represents the proportion of participants within the corresponding category, with darker shading indicating a higher prevalence. (B) Combined distribution of periodontitis grades (A–C) across stages (I–IV) after merging localized and generalized categories. Bars represent the percentage of participants in each grade for a given stage. Grade A in stages I–III was not represented due to very low percentages.

**FIGURE 2:**
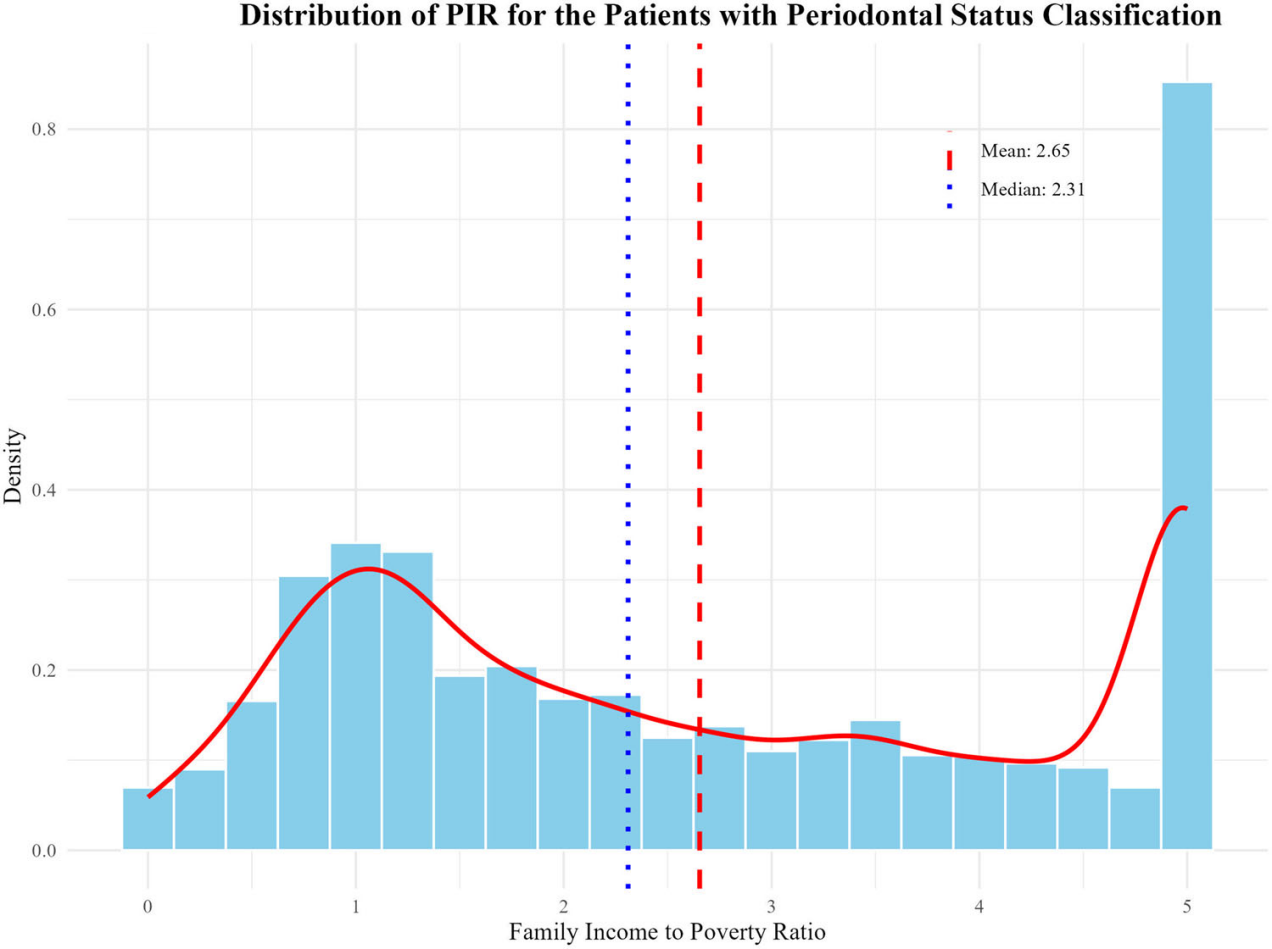
Distribution of family income‐to‐poverty ratio (PIR) among participants with a periodontal status classification. The histogram with kernel density overlay shows the spread of PIR values, with the mean (2.65) marked by a red dashed line and the median (2.31) marked by a blue dotted line.

The NHANES 2009–2014 data contain 10,598 patients with periodontitis according to the 2018 periodontal status classification, reflecting the 133,480,406 population of the USA. The highest percentage overall occurred in localized stage II grade B periodontitis at 26.21%, followed by localized stage III grade B periodontitis at 12.30%, and generalized stage II grade B periodontitis at 10.60% (Figure 1A, B). The kernel density plot (Figure 2) shows that patients with periodontal disease span various socioeconomic levels by PIR distribution.

The PIR (a proxy for higher socioeconomic status) demonstrated a consistent inverse relationship with periodontal disease severity across all classification parameters (Table 1). Higher socioeconomic status was associated with a significantly reduced risk of advanced periodontitis. The protective effect was most pronounced for disease stage, with each unit increase in PIR corresponding to 42%, 27%, and 13% reductions in the odds of stage IV (OR = 0.58), III (OR = 0.73), and II (OR = 0.87) versus stage I periodontitis, respectively (all *p* < 0.001). Similar patterns were observed for disease grade, with PIR increases associated with 26% and 17% reductions in the odds of grade C (OR = 0.74) and B (OR = 0.83) versus grade A (both *p* < 0.001). The association with disease extent showed a 7% reduction in the odds of generalized versus localized periodontitis (OR = 0.93, *p *< 0.001).

**TABLE 1:** Association of poverty‐income ratio with periodontitis stage, grade, and extent (stage I, grade A, and localized as reference).

Comparison	Odds Ratio (OR)
Stage II vs. stage I	0.87 (95% CI: 0.84–0.91, *p* < 0.001)
Stage III vs. stage I	0.73 (0.70–0.76, *p* < 0.001)
Stage IV vs. stage I	0.58 (0.55–0.61, *p* < 0.001)
Grade B vs. grade A	0.83 (0.78–0.88, *p* < 0.001)
Grade C vs. grade A	0.74 (0.70–0.79, *p* < 0.001)
Generalized vs. localized	0.93 (0.91–0.95, *p* < 0.001)

**FIGURE 3:**
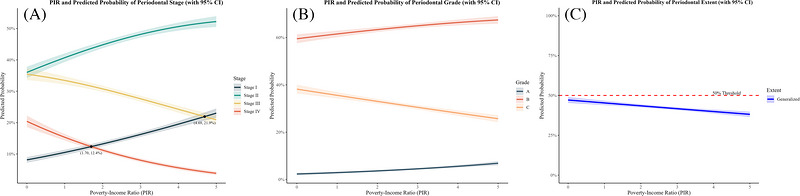
Predicted probabilities of periodontal stage (A), grade (B), and extent (C) across the range of poverty‐income ratio (PIR) values. Shaded areas represent 95% confidence intervals. (A) A higher PIR was associated with an increased predicted probability of stage I, decreased predicted probability of stages III and IV, and a gradual increase in stage II probability; (B) A higher PIR was associated with a slightly increased predicted probability of grade A, a decreased probability of grade B, and a slight increase in grade C; and (C) A higher PIR was associated with a linear decrease in the predicted probability of generalized periodontitis extent, remaining below the 50% threshold.

The PIR showed significant associations with periodontal outcomes. A higher PIR was associated with increased stage I–II periodontitis and decreased stage III–IV periodontitis, indicating that a higher income correlates with less severe disease. Stage II had the highest probability overall among relatively lower socioeconomic status (Figure 3A). For periodontal grade, grade B remained most prevalent across all income levels, grade C was second but declined with higher PIR, and grade A remained the least common (Figure 3B). Higher socioeconomic status was also associated with reduced generalized periodontal disease (Figure 3C).

The dataset consists of 56% never smokers, 25% former smokers, and 19% current smokers. There is a clear, consistent trend across all three outcomes (stage, grade, extent) between smoking and periodontitis (Table 2, Figure 4A–C). Current smokers have the highest proportion of severe outcomes (stage IV, grade C, generalized extent), while never smokers have the highest proportion of milder outcomes. Current smokers are consistently associated with elevated risks across multiple periodontal outcomes. Compared with never smokers, current smokers exhibit a 119% and 282% increased relative risk of stage III (RRR = 2.19, *p* < 0.001) and stage IV (RRR = 3.82, *p* < 0.001), and significantly higher risks of grade B and grade C, with relative risks increased by 57% (RRR = 1.57 *p* < 0.001) and 118% (RRR = 2.18, *p* < 0.001), respectively, compared with never smokers. Compared with never smokers, the odds of having generalized periodontitis are 46% higher for current smokers (OR = 1.46, *p* < 0.001) and not significantly higher for former smokers (*p* = 0.24). Across all disease stages, PIR demonstrates significant protective effects (all *p *< 0.001), with the strongest protection observed at the most severe stage (stage IV), showing a 39% risk reduction per unit increase (RRR = 0.61, *p* < 0.001). Across all stages and grades, a higher PIR was consistently protective, reducing the likelihood of more severe stages and aggressive grades. Compared with stage I, each unit increase in PIR was associated with a 12% risk reduction for stage II, 25% for stage III, and 39% for stage IV (all *p* < 0.001). Similarly, PIR was associated with a 16% risk reduction for grade B and 23% for grade C (both *p* < 0.001) compared with grade A. A minor protective effect by PIR was observed with the disease extent (OR = 0.95, *p* < 0.001).

**TABLE 2:** Association of smoking status, HbA1c, and BMI and PIR with periodontitis stage, grade, and extent (stage I, grade B, and localized as reference).

Smoking status & PIR			
Comparison	Smoking RRR	PIR RRR	
Stage II vs. I	Former smoker vs. never smoker: 1.30 (95% CI: 1.13–1.51, *p* < 0.001)	0.88 (0.85–0.91, *p* < 0.001)	
	Current smoker vs. never smoker: 1.20 (1.00‐1.44, *p* < 0.001)		
Stage III vs. I	Former smoker vs. never smoker: 1.94 (1.66–2.27, *p* < 0.001)	0.75 (0.72–0.78, *p* < 0.001)	
	Current smoker vs. never smoker: 2.19 (1.81‐2.64, *p *< 0.001)		
Stage IV vs. I	Former smoker vs. never smoker: 3.38 (2.76–4.14, *p* < 0.001)	0.61 (0.57–0.64, *p* < 0.001)	
	Current smoker vs. never smoker: 3.82 (3.05–4.78, *p* < 0.001)		
Grade B vs. A	Former smoker vs. never smoker: 1.33 (1.04–1.69, *p* = 0.022)	0.84 (0.79–0.89, *p* < 0.001)	
	Current smoker vs. never smoker: 1.57 (1.14–2.16, *p* = 0.006)		
Grade C vs. A	Former smoker vs. never smoker: 1.67 (1.30–2.14, *p* < 0.001)	0.77 (0.72–0.82, *p* < 0.001)	
	Current smoker vs. never smoker: 2.18 (1.58–3.03, *p* < 0.001)		

Comparison	Smoking OR	PIR OR	
Generalized vs. localized	Former smoker vs. never smoker: 1.06 (0.96–1.17, *p* = 0.24)	0.95 (0.92–0.97, *p* < 0.001)	
	Current smoker vs. never smoker: 1.46 (1.31–1.63, *p* < 0.001)		

HbA1c & PIR			
Comparison	HbA1c RRR	PIR RRR	
Stage II vs. I	1.56 (95% CI: 1.41–1.73, *p* < 0.001)	0.88 (0.85–0.92, *p* < 0.001)	
Stage III vs. I	1.97 (1.78–2.19, *p* < 0.001)	0.74 (0.71–0.77, *p* < 0.001)	
Stage IV vs. I	2.14 (1.92–2.38, *p* < 0.001)	0.59 (0.56–0.63, *p* < 0.001)	
Grade B vs. A	1.58 (1.33–1.88, *p* < 0.001)	0.84 (0.79–0.89, *p* < 0.001)	
Grade C vs. A	1.82 (1.52–2.17, *p* < 0.001)	0.76 (0.71–0.81, *p* < 0.001)	

Generalized vs. localized	OR		
HbA1c	1.00 (0.97–1.04, *p* = 0.81)		
PIR	0.93 (0.91–0.95, *p* < 0.001)		

**BMI & PIR**			
Comparison	BMI RRR	PIR RRR	Interaction (BMI × PIR)
Stage II vs. I	0.98 (95% CI: 0.97–1.00, *p* = 0.041)	0.69 (0.59–0.81, *p* < 0.001)	1.01 (1.00–1.01, *p* = 0.003)
Stage III vs. I	0.97 (0.95–0.98, *p* < 0.001)	0.48 (0.41–0.58, *p* < 0.001)	1.01 (1.01–1.02, *p* < 0.001)
Stage IV vs. I	0.93 (0.91–0.95, *p* < 0.001)	0.30 (0.23–0.39, *p* < 0.001)	1.02 (1.01–1.03, *p* < 0.001)
Grade B vs. A	0.97 (0.94–0.99, *p* = 0.014)	0.54 (0.42–0.71, *p* < 0.001)	1.01 (1.00–1.02, *p* < 0.001)
Grade C vs. A	0.95 (0.93–0.98, *p* < 0.001)	0.43 (0.33–0.56, *p* < 0.001)	1.02 (1.01–1.03, *p* < 0.001)

Generalized vs. localized	OR		
BMI	1.00 (0.99–1.00 *p* = 0.184)		
PIR	0.93 (0.90–0.95, *p* < 0.001)		

Abbreviation: BMI, body mass index; OR, odds ratio; PIR, poverty‐income ratio; RRR, relative risk ratio.

FIGURE 4(A) Predicted probability of each periodontal stage (I–IV) across the range of poverty‐to‐income ratio (PIR) values, stratified by smoking status (never smoker, former smoker, current smoker). Never smokers consistently show higher probabilities for stages I and II and lower probabilities for stages III and IV compared with smokers. (B) Predicted probability of periodontal grades (A–C) across the range of poverty‐to‐income ratio (PIR) values, stratified by smoking status (never smoker, former smoker, current smoker). Never smokers show slightly higher probabilities for grades A and B and lower probabilities for grade C compared with smokers. (C) Predicted probability of generalized periodontal extent across the range of poverty‐to‐income ratio (PIR) values, stratified by smoking status (never smoker, former smoker, current smoker). A higher PIR is associated with a consistent decrease in predicted probability across all smoking categories, with current smokers showing the highest probabilities.

**Figure:**
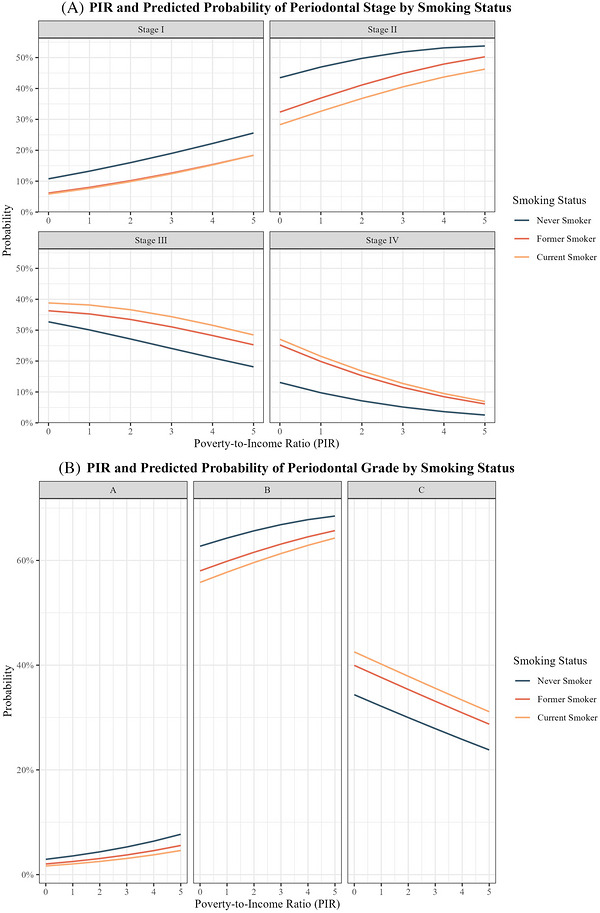

**Figure:**
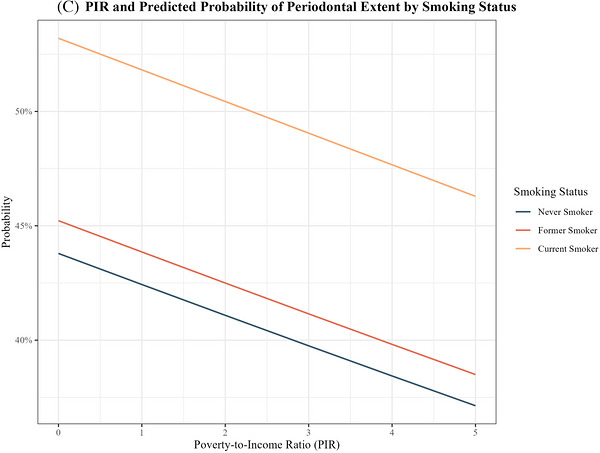

Higher HbA1c levels and lower PIR are progressively associated with more severe periodontitis stages and grades (Table 2). When the PIR was held constant, each unit increase in HbA1c significantly elevated the risk of advancing to higher disease stages, with relative risk ratios of 1.56, 1.97, and 2.14 for stages II, III, and IV, respectively, compared with stage I (all *p* < 0.001). Conversely, when HbA1c was controlled, PIR demonstrated progressively stronger protective associations across disease stages, with stage IV exhibiting an RRR of 0.59 compared with stage I (*p *< 0.001), corresponding to a 41% risk reduction. Similar patterns emerged for periodontal grading, with HbA1c showing RRRs of 1.58 and 1.82 for grades B and C compared with grade A when PIR was held constant, while PIR demonstrated protective effects (RRRs of 0.84 and 0.76) representing 16%–24% risk reduction relative to grade A when HbA1c was controlled (all *p* < 0.001). Regarding the periodontitis extent, HbA1c showed no significant association with generalized versus localized disease (OR = 1.00, *p* = 0.81), while PIR remained significantly but minimally protective (OR = 0.93, *p* < 0.001).

A lower BMI and lower income ratio are progressively associated with more severe periodontitis stages and grades (Table 2). A statistically significant BMI–PIR interaction was retained, though the effect size was modest. A higher BMI demonstrated slight protective associations across disease stages and grades, with RRR consistently ranging from 0.93 to 0.98. The PIR demonstrated substantially stronger protective associations when BMI was held constant. For stage, the progression is significantly linear and dose‐dependent with PIR: each unit increase in PIR reduces the risk of stage II by 31% (RRR = 0.69, *p* < 0.001), the risk of stage III by 52% (RRR = 0.48, *p* < 0.001), and the risk of stage IV by a remarkable 70% (RRR = 0.30, *p* < 0.001), compared with stage I. For grade, the progressive pattern of PIR is less pronounced, but the protective effect remains significant, with a 46% risk reduction for grade B (RRR = 0.54, *p* < 0.001) and a 57% risk reduction for grade C (RRR = 0.43, *p* < 0.001) compared with grade A. For extent, BMI was not associated with disease extent (OR = 1.00, *p* = 0.184), whereas PIR showed a weak but significant protective effect against generalized periodontitis (OR = 0.93, *p* < 0.001).

## 4 DISCUSSION

This study shows the distribution of periodontitis NHANES data from 2009 to 2014 by stage, grade, and extent. We report that higher socioeconomic status, indicated by PIR, is a significant protective factor against periodontal disease severity, progression, and distribution. The analysis revealed dose‐dependent protective effects of a higher PIR across disease stage, grade, and extent, with the strongest protection observed for stage IV and grade C periodontitis (39% and 23% risk reduction per unit PIR increase, respectively). Smoking emerged as a critical risk factor, with current smokers exhibiting up to a 282% increased relative risk for stage IV disease and a 118% increased risk for grade C periodontitis compared with never smokers. Additionally, higher HbA1c levels consistently elevated disease severity risk, with a relative risk ratio reaching 2.14 for stage IV disease, while higher BMI demonstrated slight protective associations across disease stages and grades.

This study has several notable features. First, we utilized the NHANES dataset, which provides nationally representative data that allows for population‐level inferences about periodontal health across the US. This approach may help to address some limitations of previous investigations that have relied on smaller samples when examining risk factors within the new periodontitis classification framework.^8^, ^26^, ^27^ Second, while prior research has predominantly focused on periodontitis prevalence,^8^, ^26^ our investigation examined how risk factors associate with disease severity levels, progression patterns, and overall distribution. This was facilitated by implementing the ACES framework that allowed us to assign patient‐level stage, grade, and extent.^7^ Where previous NHANES studies could only report on disease stage, mostly without being able to distinguish between stage III and IV,^28^ this approach may contribute to a more detailed understanding of periodontal disease manifestation. Third, our analytical framework considered both socioeconomic determinants and established clinical risk factors, allowing for examination of their independent and combined effects on periodontal outcomes. This modeling approach attempts to capture some of the complex relationships between social and biological factors that may influence periodontal health.

Socioeconomic status demonstrates a strong inverse association with periodontitis prevalence across diverse populations.^12^ The global burden of disease analyses reveal higher rates of severe periodontitis among individuals with lower socioeconomic indicators,^9^, ^29^, ^30^ while NHANES data (2009–2014) showed twice the periodontitis prevalence among low‐income versus higher‐income adults in the US.^2^ Further NHANES analysis (2011–2018) suggests that 50% reductions in absolute and relative poverty could decrease the periodontitis prevalence by 4% and 12%, respectively.^31^ Our findings strongly corroborate and extend these patterns by providing detailed quantitative insights into the protective effects of PIR against periodontal disease severity and progression. Each one‐unit increase in PIR corresponded to a 13%–42% decrease in the odds of a higher periodontitis stage, 17%–26% decrease for higher grade, and 7% decrease for generalized periodontitis. These protective effects became more pronounced in multivariable analyses, with each PIR unit increase associated with a 39%–70% reduction in stage IV risk versus stage I and a 23%–57% reduction in grade C risk versus grade A. These findings translate to meaningful clinical implications for patient care. Patients living below the poverty line (PIR < 1.0) demonstrated a substantially elevated periodontal disease risk, warranting intensified preventive care and more frequent monitoring. This threshold aligns with eligibility for government insurance programs, which typically cover preventive services but exclude active periodontal treatment and maintenance therapy.^32^ For clinicians, socioeconomic status should be integrated into periodontal risk assessment as a key indicator comparable to smoking or diabetes status.^33^ In patient counseling, these data support direct conversations about how financial constraints may limit access to regular maintenance care and increase the risk of disease progression.^34^ Resource navigation becomes an essential component of comprehensive periodontal care for low PIR populations. From a public health perspective, expanding insurance coverage to include periodontal maintenance therapy for high‐risk, low‐income populations represents a cost‐effective intervention, as regular maintenance prevents disease progression that ultimately requires more expensive treatment.^35^ The magnitude of these protective effects demonstrates that periodontal health disparities reflect broader social inequities with associated risk factors.

Since periodontitis shares common risk factors with other NCDs,^36^ these findings align with WHO priorities for enhancing global oral health by 2030.^37^ This WHO initiative supports the organization's commitment to universal health coverage through equitable healthcare access without imposing financial hardship, while simultaneously targeting rising rates of NCDs that share common risk factors with periodontal conditions. However, addressing oral health disparities effectively, particularly within high‐income countries such as the US, requires comprehensive policy approaches that transcend poverty‐focused interventions alone.^38^ Societal living circumstances represent fundamental drivers of health disparities across populations,^39^ and detrimental health behaviors such as smoking among people in lower socioeconomic positions often represent adaptive responses to challenging life circumstances.^40^ Multifaceted approaches to defining economic hardship and resource limitations, which incorporate social factors affecting oral health, are essential for achieving meaningful population health improvements. Our study's detailed exploration of PIR associations with periodontal risk factors provides evidence‐based foundations for tailored clinical interventions and targeted public health strategies. This enables both clinicians and policymakers to address the fundamental social conditions that drive oral health inequalities through patient‐centered care approaches and context‐appropriate community interventions^38^

This study also confirms the well‐established relationship between smoking and periodontal disease severity. The observed pattern where current smokers exhibit the highest proportion of severe outcomes (stage IV, grade C, generalized extent) while never smokers show predominantly milder outcomes reinforces earlier findings that smoking may be responsible for more than half of periodontitis cases among adults in the US.^17^ Current smokers demonstrated substantially elevated risks across multiple disease measures, showing a 119% and 282% increased relative risks for stage III and IV disease, respectively, and 57% and 118% increased risks for grade B and C progression. These findings provide clear evidence of the detrimental impact of smoking on periodontal health outcomes^41^ and align with the BigMouth study results confirming smoking's association with rapid progression (grade C) of periodontal disease.^42^, ^43^ The 46% higher odds of generalized periodontitis among current smokers further establish the understanding that smoking not only increases disease susceptibility but also promotes more extensive and severe manifestations of periodontal disease.^17^ While potential confounding factors warrant further investigation, these findings collectively reinforce the clinical importance of smoking cessation interventions, as previous research has demonstrated both smoking's adverse influence on treatment outcomes^44^ and the positive effects of smoking cessation on periodontitis occurrence and periodontal healing.^43^

The relationship between diabetes and periodontitis represents a significant clinical concern, with glycemic control serving as a critical determinant of periodontal health outcomes. Previous epidemiological studies have consistently demonstrated that poorly controlled diabetes significantly elevates periodontitis risk, with NHANES data (1988‐1994) showing an approximately threefold increased susceptibility among individuals with HbA1c > 9%.^45^ Meta‐analyses of adjusted estimates further support this relationship, indicating an 86% increased risk of periodontitis incidence or progression in diabetic patients.^46^ Longitudinal evidence has documented that uncontrolled diabetes correlates with accelerated periodontal destruction, manifesting as a 0.35 mm additional attachment loss over 5 years.^47^ More recent investigations in Norway using the 2018 classification system have confirmed that patients with HbA1c > 6.5% face more than double the risk of progressing to advanced periodontal stages.^48^ Consistent with these established findings, our analysis demonstrated that after adjusting for socioeconomic factors, each unit increase in HbA1c conferred a 1.5–2.1 fold increased risk of progression to severe periodontal stages and grades. This finding reinforces the progressive dose–response relationship between glycemic control and periodontal disease severity. These findings underscore the clinical imperative for integrated diabetes‐periodontal care, suggesting that optimizing glycemic control should be prioritized as a fundamental component of periodontal disease prevention and management strategies.

Obesity is recognized as an important risk factor for periodontal disease development and progression through chronic inflammation and metabolic dysfunction.^49^, ^50^ Previous studies have consistently shown positive associations between elevated BMI and periodontitis severity, with NHANES (2011–2014) data demonstrating a 1% increase in periodontitis incidence per unit BMI increase.^51^ Additionally, a Turkish study found that individuals with a BMI > 25 had an approximately four‐fold higher risk of stage II–IV periodontitis compared with those with BMI < 25.^52^ In contrast, our analysis revealed slight protective associations between a higher BMI and both periodontal stages and grades under same PIR, with consistent risk reductions of 2%–7% across severity categories. This counterintuitive finding may reflect a non‐linear relationship between BMI and periodontitis that is modified by unmeasured socioeconomic factors, or alternatively, survival bias and selective dental care seeking behaviors among individuals with a higher BMI within poverty groups. Future stratified analyses are needed to clarify these complex relationships. These findings suggest that BMI alone may not reliably predict periodontitis severity across socioeconomically diverse populations, emphasizing the need for comprehensive risk assessment.

Several limitations should be acknowledged in this study. First, this study utilized the PIR as the sole socioeconomic indicator. While correlated, different socioeconomic measures capture distinct dimensions with varying health associations, potentially limiting our understanding of socioeconomic effects.^12^ Second, the analysis did not consider other confounding factors including age, sex, race/ethnicity, systemic diseases beyond diabetes, and oral hygiene practices, which may have introduced unmeasured confounding and affected generalizability. Third, although vertical defects and furcation are important for staging criteria, this information was not available in NHANES data, potentially affecting periodontal disease classification. Fourth, this study utilized NHANES 2009‐2014 data, the most recent cycle with comprehensive full‐mouth periodontal examinations available. However, demographic shifts, healthcare policy changes, and evolving socioeconomic patterns over the past decade may affect the current applicability of these findings. Future studies incorporating additional confounding variables with newer comprehensive periodontal data would help to confirm these associations in contemporary populations.

## 5 CONCLUSIONS

In NHANES 2009–2014 cycles with adults classified by the 2018 system according to the ACES framework, a higher PIR was associated with lower periodontitis stage, grade, and generalized extent. Current smoking status was associated with higher stage, grade, and greater odds of generalized disease, while former smoking was not significantly associated with extent. Higher HbA1c was associated with higher stage and grade, with no association with extent. BMI showed small inverse associations with stage and grade and also no association with extent.

## AUTHOR CONTRIBUTIONS

**Meng Xuan Chen**: Involved in conceptualization; methodology; formal analysis; investigation; writing the original draft; reviewing; editing. **Yue Yu**: Involved in methodology; software; validation; formal analysis. **Chen Xuan Wei**: Involved in reviewing; editing. **Hamoun Sabri**: Involved in data collection. **Muhammad H. A. Saleh**: Involved in methodology; investigation; data curation; validation; reviewing; editing.

## CONFLICT OF INTEREST STATEMENT

The authors declare no conflicts of interest in this study.

## Data Availability

The data that support the findings of this study are available in National Health and Nutrition Examination Survey at https://wwwn.cdc.gov/nchs/nhanes/nhanes3/datafiles.aspx. These data were derived from the following resources available in the public domain: 2009–2010; 2011–2012; and 2013–2014, https://wwwn.cdc.gov/nchs/nhanes/nhanes3/datafiles.aspx

## References

[1] Bernabe E , Marcenes W , Abdulkader RS , et al. Trends in the global, regional, and national burden of oral conditions from 1990 to 2021: a systematic analysis for the global burden of disease study 2021. The Lancet. 2025;405:897‐910. 10.1016/S0140-6736(24)02811-3 40024264

[2] Eke PI , Thornton‐Evans GO , Wei L , Borgnakke WS , Dye BA , Genco RJ . Periodontitis in US adults: national health and nutrition examination survey 2009‐2014. J Am Dent Assoc. 2018;149:576‐588. e576. 29957185 10.1016/j.adaj.2018.04.023 PMC8094373

[3] Jin L , Lamster I , Greenspan J , Pitts N , Scully C , Warnakulasuriya S . Global burden of oral diseases: emerging concepts, management and interplay with systemic health. Oral Dis. 2016;22:609‐619. 26704694 10.1111/odi.12428

[4] Eke PI , Wei L , Thornton‐Evans GO , et al. Risk indicators for periodontitis in US adults: nHANES 2009 to 2012. J Periodontol. 2016;87:1174‐1185. 27367420 10.1902/jop.2016.160013 PMC11370315

[5] Papapanou PN , Sanz M , Buduneli N , et al. Periodontitis: consensus report of workgroup 2 of the 2017 world workshop on the classification of periodontal and peri‐implant diseases and conditions. J Periodontol. 2018;89:S173‐S182. 29926951 10.1002/JPER.17-0721

[6] Ravidà A , Qazi M , Troiano G , et al. Using periodontal staging and grading system as a prognostic factor for future tooth loss: a long‐term retrospective study. J Periodontol. 2020;91:454‐461. 31502244 10.1002/JPER.19-0390

[7] Holtfreter B , Kuhr K , Borof K , et al. ACES: a new framework for the application of the 2018 periodontal status classification scheme to epidemiological survey data. J Clin Periodontol. 2024;51:512‐521. 38385950 10.1111/jcpe.13965

[8] Tay JRH , Holtfreter B , Baumeister SE , Peres MA , Nascimento GG . Application of the 2018 periodontal status classification to epidemiological survey data (ACES) framework to estimate the periodontitis prevalence in the United States. J Clin Periodontol. 2025;52:1032‐1043. 39895381 10.1111/jcpe.14132 PMC12176459

[9] Chen MX , Zhong YJ , Dong QQ , Wong HM , Wen YF . Global, regional, and national burden of severe periodontitis, 1990‐2019: an analysis of the global burden of disease study 2019. J Clin Periodontol. 2021;48:1165‐1188. 34101223 10.1111/jcpe.13506

[10] Borrell LN , Crawford ND . Social disparities in periodontitis among United States adults 1999‐2004. Community Dent Oral Epidemiol. 2008;36:383‐391. 18924254 10.1111/j.1600-0528.2007.00406.x

[11] Sabbah W , Tsakos G , Chandola T , Sheiham A , Watt R . Social gradients in oral and general health. J Dent Res. 2007;86:992‐996. 17890677 10.1177/154405910708601014

[12] Borrell LN , Crawford ND . Socioeconomic position indicators and periodontitis: examining the evidence. Periodontol 2000. 2012;58:69‐83. 22133367 10.1111/j.1600-0757.2011.00416.x PMC3233193

[13] Garrett BE , Martell BN , Caraballo RS , King BA . Socioeconomic differences in cigarette smoking among sociodemographic groups. Prev Chronic Dis. 2019;16:E74. 31198164 10.5888/pcd16.180553 PMC6583815

[14] Sheets L , Petroski GF , Jaddoo J , et al. The effect of neighborhood disadvantage on diabetes prevalence. AMIA Annu Symp Proc. 2018;2017:1547. 29854224 PMC5977699

[15] Anekwe CV , Jarrell AR , Townsend MJ , Gaudier GI , Hiserodt JM , Stanford FC . Socioeconomics of obesity. Curr Obes Rep. 2020;9:272‐279. 32627133 10.1007/s13679-020-00398-7 PMC7484407

[16] Von Elm E , Altman DG , Egger M , Pocock SJ , Gøtzsche PC , Vandenbroucke JP . The strengthening the reporting of observational studies in epidemiology (STROBE) statement: guidelines for reporting observational studies. The Lancet. 2007;370:1453‐1457. 10.1016/S0140-6736(07)61602-X 18064739

[17] Tomar SL , Asma S . Smoking‐attributable periodontitis in the United States: findings from NHANES III. J Periodontol. 2000;71:743‐751. 10.1902/jop.2000.71.5.743 29537517

[18] Hsu YT , Huang NC , Wong A , et al. Periodontal risk assessment based on dental and gingival morphology: a comparative analysis of African versus Asian American cohorts. Clin Adv Periodontics. 2020;10:224‐230. 32717138 10.1002/cap.10117

[19] Jayawardena CK , Abesundara AP , Nanayakkara DC , Chandrasekara MS . Age‐related changes in crown and root length in Sri Lankan Sinhalese. J Oral Sci. 2009;51:587‐592. 20032612 10.2334/josnusd.51.587

[20] Nesse W , Abbas F , Van Der Ploeg I , Spijkervet FKL , Dijkstra PU , Vissink A . Periodontal inflamed surface area: quantifying inflammatory burden. J Clin Periodontol. 2008;35:668‐673. 18564145 10.1111/j.1600-051X.2008.01249.x

[21] Winkler P , Dannewitz B , Nickles K , Petsos H , Eickholz P . Assessment of periodontitis grade in epidemiological studies using interdental attachment loss instead of radiographic bone loss. J Clin Periodontol. 2022;49:854‐861. 35713218 10.1111/jcpe.13679

[22] Capitaneanu C , Willems G , Jacobs R , Fieuws S , Thevissen P . Sex estimation based on tooth measurements using panoramic radiographs. Int J Legal Med. 2017;131:813‐821. 27534562 10.1007/s00414-016-1434-0

[23] Salonen LW , Frithiof L , Wouters FR , Helldén LB . Marginal alveolar bone height in an adult Swedish population: a radiographic cross‐sectional epidemiologic study. J Clin Periodontol. 1991;18:223‐232. 1856302 10.1111/j.1600-051x.1991.tb00419.x

[24] Minhas AMK , Jain V , Li M , et al. Family income and cardiovascular disease risk in American adults. Sci Rep. 2023;13:279. 36609674 10.1038/s41598-023-27474-x PMC9822929

[25] Yi H , Li M , Dong Y , et al. Nonlinear associations between the ratio of family income to poverty and all‐cause mortality among adults in NHANES study. Sci Rep. 2024;14:12018. 38797742 10.1038/s41598-024-63058-z PMC11128441

[26] Stødle IH , Verket A , Høvik H , Sen A , Koldsland OC . Prevalence of periodontitis based on the 2017 classification in a Norwegian population: the HUNT study. J Clin Periodontol. 2021;48:1189‐1199. 34101228 10.1111/jcpe.13507

[27] Dukka H , Dietrich T , Saleh MH , et al. Prognostic performance of the 2017 World Workshop classification on staging and grading of periodontitis compared with the British Society of Periodontology's implementation. J Periodontol. 2022;93:537‐547. 34314515 10.1002/JPER.21-0296

[28] Saleh MH , Kalani K , Sabri H , et al. Association between periodontitis severity and prostate‐specific antigen levels using the NHANES data. J Periodontol. 2025;96(10):1113‐1125. 40099890 10.1002/JPER.24-0561 PMC12572693

[29] Ghanem AS , Bata R , Kovács N , Nagy AC . Sociodemographic inequalities in the global burden trends and machine learning‐based projections of periodontitis from 1990 to 2030 across different development levels. Front Oral Health. 2025;6:1609961. 40599686 10.3389/froh.2025.1609961 PMC12209297

[30] Saleh MH , Dias DR , Kumar P . The economic and societal impact of periodontal and peri‐implant diseases. Periodontol 2000. 2024. 10.1111/prd.12568 PMC12842886 38693603

[31] Cooray U , Singh A , Aida J , Tsakos G , Peres M . Impact of poverty reduction on oral health outcomes among US adults. J Dent Res. 2025;104(10):1069‐1076. 40326603 10.1177/00220345251323183

[32] Canonico M . Medicaid adult dental benefits coverage by state. Center for Health Care Strategies, Inc. 2019:1‐6.

[33] Darby I . Risk factors for periodontitis & peri‐implantitis. Periodontol 2000. 2022;90:9‐12. 35913624 10.1111/prd.12447 PMC9804916

[34] Willems S , De Maesschalck S , Deveugele M , Derese A , De Maeseneer J . Socio‐economic status of the patient and doctor–patient communication: does it make a difference?. Patient Educ Couns. 2005;56:139‐146. 15653242 10.1016/j.pec.2004.02.011

[35] Pattamatta M , Chapple I , Listl S . The value‐for money of preventing and managing periodontitis: opportunities and challenges. Periodontol 2000. 2024. 10.1111/prd.12569 PMC12842866 38745388

[36] Wolf TG , Cagetti MG , Fisher J‐M , Seeberger GK , Campus G . Non‐communicable diseases and oral health: an overview. Front Oral Health. 2021;2:725460. 35048049 10.3389/froh.2021.725460 PMC8757764

[37] Organization WH . Global oral health status report: towards universal health coverage for oral health by 2030. World Health Organization; 2022.

[38] Tsakos G , Watt RG , Guarnizo‐Herreño CC . Reflections on oral health inequalities: theories, pathways and next steps for research priorities. Community Dent Oral Epidemiol. 2023;51:17‐27. 36744970 10.1111/cdoe.12830

[39] Freudenberg N , Franzosa E , Chisholm J , Libman K . New approaches for moving upstream: how state and local health departments can transform practice to reduce health inequalities. Health Educ Behav. 2015;42:46S‐56S. 25829117 10.1177/1090198114568304

[40] Nettle D . Why are there social gradients in preventative health behavior? A perspective from behavioral ecology. PLoS One. 2010;5:e13371. 20967214 10.1371/journal.pone.0013371 PMC2954172

[41] Ravidà A , Saleh MH , Ghassib IH , et al. Impact of smoking on cost‐effectiveness of 10–48 years of periodontal care. Periodontology. 2000:2024. 10.1111/prd.12585 PMC12842896 39054672

[42] Saleh MH , Sabri H . Dose‐dependent association of systemic comorbidities with periodontitis severity: a large population cross‐sectional study. J Periodontol. 2025. 10.1002/JPER.25-0055 PMC13001135 40778524

[43] Chatzopoulos GS , Jiang Z , Marka N , Wolff LF . Association between periodontitis extent, severity, and progression rate with systemic diseases and smoking: a retrospective study. J Pers Med. 2023;13:814. 37240984 10.3390/jpm13050814 PMC10223170

[44] Nociti Jr FH , Casati MZ , Duarte PM . Current perspective of the impact of smoking on the progression and treatment of periodontitis. Periodontol 2000. 2015;67:187‐210. 25494601 10.1111/prd.12063

[45] Tsai C , Hayes C , Taylor GW . Glycemic control of type 2 diabetes and severe periodontal disease in the US adult population. Community Dent Oral Epidemiol. 2002;30:182‐192. 12000341 10.1034/j.1600-0528.2002.300304.x

[46] Alwithanani N . Periodontal diseases and diabetes mellitus: a systematic review. J Pharm Bioallied Sci. 2023;15:S54‐S63. 37654263 10.4103/jpbs.jpbs_515_22 PMC10466651

[47] Demmer RT , Holtfreter B , Desvarieux M , et al. The influence of type 1 and type 2 diabetes on periodontal disease progression: prospective results from the study of health in pomerania (SHIP). Diabetes Care. 2012;35:2036‐2042. 22855731 10.2337/dc11-2453 PMC3447825

[48] Stødle IH , Sen A , Høvik H , Verket A , Koldsland OC . Association between periodontitis stages and self‐reported diseases in a Norwegian population: the HUNT study. BMC Oral Health. 2023;23:999. 38093278 10.1186/s12903-023-03743-z PMC10720083

[49] Nascimento GG , Leite FRM , Do LG , et al. Is weight gain associated with the incidence of periodontitis? A systematic review and meta‐analysis. J Clin Periodontol. 2015;42:495‐505. 25952821 10.1111/jcpe.12417

[50] Suvan J , D'Aiuto F , Moles DR , Petrie A , Donos N . Association between overweight/obesity and periodontitis in adults. A systematic review. Obes Rev. 2011;12:e381‐e404. 21348914 10.1111/j.1467-789X.2010.00808.x

[51] Liu L , Xia LY , Gao YJ , Dong XH , Gong RG , Xu J . Association between obesity and periodontitis in US adults: NHANES 2011–2014. Obesity Facts. 2023:1‐12. 10.1159/000534751 PMC10836934 37935140

[52] Çetin MB , Sezgin Y , Önder C , Bakirarar B . The relationship between body mass index and stage/grade of periodontitis: a retrospective study. Clin Oral Investig. 2022;26:1937‐1945. 10.1007/s00784-021-04172-4 34709456

